# Liver and spleen of hosts of *Rhipicephalus linnaei* exposed to synthetic (afoxolaner) and natural acaricides (esters from castor oil). A comparative clinical-morphological study

**DOI:** 10.1590/S1984-29612023041

**Published:** 2023-07-17

**Authors:** Luís Fernando Sodelli, Odaiza da Silva, Bruna Jéssyca Nascimento Araújo, Maria Izabel Camargo-Mathias

**Affiliations:** 1 Departamento de Biologia Geral e Aplicada, Instituto de Biociências, Universidade Estadual Paulista “Júlio de Mesquita Filho” – UNESP, Rio Claro, SP, Brasil

**Keywords:** Brown dog tick, natural product, Ricinus communis, isoxazoline, Nexgard®, Carrapato vermelho do cão, produto natural, Ricinus communis, isoxazolina, Nexgard®

## Abstract

In dogs, *Rhipicephalus linnaei* transmits pathogens such as *Ehrlichia canis, Babesia vogeli*, and *Hepatozoon cani*s. The veterinary market has synthetic acaricides to ticks control. Esters derived from castor oil are efficient. However, there is little information about their effects on non-target organisms. This work consisted of a clinical (AST, ALT, and ALP) and histological and histochemical analysis (liver and spleen) of female rabbits exposed to these esters and afoxolaner. The rabbits were divided into three groups: control group (CG) received Bandeirante® rabbit feed; the afoxolaner treatment (TG1) received rabbit feed and two doses of afoxolaner; castor oil esters treatment (TG2) received rabbit feed enriched with esters (1.75 g esters/kg). No alterations were observed in the AST, ALT, and ALP enzymes in exposure to esters TG2. Rabbits from TG1 showed changes in AST. The liver of rabbits exposed to afoxolaner underwent histological and histochemical changes, such as steatosis and vacuolation, as well as poor protein labeling. Polysaccharides were intensely observed in the group exposed to esters. The spleen showed no changes in any of the exposure. Esters from castor oil caused fewer liver changes when incorporated into the feed and fed to rabbits than exposure to afoxolaner.

## Resumo

Carrapatos *Rhipicephalus linnaei* transmitem patógenos como *Ehrlichia canis*, *Babesia vogeli* e *Hepatozoon canis,* para os cães. O mercado veterinário dispõe de acaricidas sintéticos para o controle desses ectoparasitas. Ésteres derivados do óleo de mamona são eficientes, mas há pouca informação sobre seus efeitos em organismos não-alvos. Esse trabalho objetivou analisar clínica (AST, ALT e ALP), histológica e histoquímicamente (fígado e baço) coelhas expostas aos ésteres e ao afoxolaner, tendo sido as mesmas alocadas nos grupos: Controle (GC) que recebeu ração Bandeirante®; Tratamento 1 (TG1), além da ração foram expostas ao afoxolaner, Tratamento 2 (TG2) receberam ração enriquecida com os ésteres (1,75 g ésteres/kg). Não houve alterações nas enzimas AST, ALT e ALP na exposição aos ésteres (TG2). No TG1 houve alterações na AST. O fígado das coelhas do TG1 apresentou alterações histológicas e histoquímicas, tais como esteatose e vacuolização, bem como fraca marcação de proteínas. Polissacarídeos foi intensamente marcados no fígado do TG2. O baço não apresentou alterações em nenhum dos grupos. Os ésteres do óleo de mamona causaram menos alterações hepáticas nas coelhas do que quando elas foram expostas ao afoxolaner.

## Introduction

Many mammal species are hosts to ectoparasites that frequently infest them and transmit causative agents of various diseases (Nakkoud et al., 2022). *Rhipicephalus linnaei*, formerly known as the tropical lineage of *R. sanguineus*, known as the brown dog tick, has canids as its primary hosts (Abreu et al., 2020; Dantas-Torres, 2008; Šlapeta et al., 2022). This arthropod is distributed worldwide and can transmit dangerous pathogens to dogs, such as *Ehrlichia canis*, causative agent of canine monocytic ehrlichiosis (CME). In addition, protozoa such as *Babesia vogeli* and *Hepatozoon canis* can also cause serious illness (Huggins et al., 2022; Chaber et al., 2022). The veterinary market offers a diversity of acaricides formulated from different chemical classes. Many of these products are toxic and select resistant individuals (Sunkara et al., 2022). Even with sparing use, these chemical agents can cause a wide range of problems and complications in the host of the ectoparasite, the dog. According to the information provided by the manufacturer in the package insert afoxolaner, a member of the pharmacological class of isoxazolines, episodes of adverse reactions such as vomiting, dry skin or peeling, skin irritation (itching), diarrhea, lethargy, and anorexia have manifested in controlled field studies. These events were classified as non-serious and rare and occurred in a self-limiting manner, of short duration, and with a reduction in subsequent doses.

Afoxolaner is one of the acaricidal medications available on the veterinary market. It protects the animal against ticks, fleas, and mites (Tinkruejeen et al., 2019) and is presented in a 0.5 g chewable form containing 11.3 mg of afoxolaner per tablet (Drag et al., 2014) and formulated from a chemical class belonging to the isoxazoline family. This drug acts on the y-aminobutyric acid (GABA) receptor and glutamate receptors, causing excessive neural stimulation and arthropod death 48 hours after the bite (Beugnet et al., 2015; Cutolo et al., 2021), acting quickly in the host organism, which is a considerable advantage. The drug acts as an acaricide for approximately one month and is recommended for dogs at 30-day intervals (Burgio et al., 2016; Beugnet et al., 2015; Tinkruejeen et al., 2019).

Medicinal plants have been used for curing diseases, preferably in humans and they are the bases of new products with acaricide action (Mabasa et al., 2021). Therefore, bioactive compounds found in plants have been the source of new studies and include esters derived from the ricinoleic acid of castor oil (*Ricinus communis*). These bioactives act as a natural acaricide against ticks, changing the morphology and physiology of organs, such as salivary glands and ovaries. Pilot studies have indicated that they would not cause damage to the hosts or the environment (Arnosti et al., 2011; Sodelli et al., 2022; Camargo-Mathias, 2018). In general, most studies on acaricidal products focus on understanding how it acts on the ectoparasite organism (Abreu et al., 2020; Souza et al., 2019; Sodelli et al., 2022), overlooking how these chemicals would act on the host systems and other non-target organisms (Camargo-Mathias, 2018). In that sance, the main objective of this study was to evaluate the effects of acaricidal substances (of natural and synthetic origin) through clinical examinations of liver enzymes and morpho-histology of the liver and spleen of tick host animals exposed to castor oil ricinoleic acid esters incorporated in the commercial feed of the brand Bandeirante® and the drug afoxolaner (chemical acaricide belonging to the isoxazoline family). These organs were chosen because they are critical indicators of the cytotoxic potential of chemical agents since the liver detoxifies the body and the spleen defends against antigens present in the blood.

## Material and Methods

### Host rabbits

Nine rabbits from the Botucatu Genetic Group, aged two months, weighing 3.0 to 3.5 kg, without previous contact with ticks or acaricides, acquired at UNESP/Botucatu (SP) were used. They were divided into three groups: GC (control group), TG1 (treatment with afoxolaner) and TG2 (treatment with castor oil). They were placed in individual cages made of galvanized wire, for adaptation, under controlled temperature and photoperiods, receiving water and food ad libitum.

### Castor oil ricinoleic acid esters (*Ricinus communis*)

To evaluate the effects of esters, the rabbits were fed 175g of ester-enriched feed daily at a concentration of 1.75 g ester/kg of Bandeirante® commercial feed, a concentration previously established in a pilot study conducted by Sodelli (2019).

## Experimental Design

1. **Control Group (CG):** the rabbits were provided daily with Bandeirante® commercial feed for 60 days.
2. **Afoxolaner treatment (TG1):** rabbits were fed Bandeirante® commercial feed and exposed to two doses in 0.5 g chewable form containing 11.3 mg afoxolaner per Nexgard® tablet for 60 days with a 30 day interval between administrations.
3. **Castor oil treatment (TG2):** the rabbits received daily the Bandeirante® commercial feed enriched with castor oil ricinoleic acid esters at a 1.75 g of esters/Kg of feed for 60 days.

## Clinical Analysis

Tables 1-3 show the results obtained from the blood samples of rabbits representatives of the three groups evaluating enzymes AST, ALT, and ALP, performed using the BIOPLUS device (semi-automatic multiparametric analyzer of colorimetric and enzymatic assays for biochemistry), LABTEST® reagents, following the protocols performed by the manufacturer.

**Table 1:** Data found in the evaluation of the enzyme aspartate aminotransferase (AST) of all animals of all groups analyzed in the present study at different times of blood collection. Note the reference values for this enzyme on the right of the table. Note (in red) the values found in the TG1 that are above those references.

**Aspartate Aminotransferase (AST)**							
**Group**	**Rabbit**	**T 0**	**T 15**	**T 30**	**T 45**	**T 60**	**Reference value**
**CG**	**1**	44	38	42	38	40	18 – 56 U/L
	**2**	33	47	44	41	39	
	**3**	34	44	39	34	41	
**TG1**	**1**	37	102	68	92	59	
	**2**	32	123	61	103	68	
	**3**	48	98	66	85	64	
**TG2**	**1**	32	34	30	29	36	
	**2**	40	36	39	43	42	
	**3**	46	40	41	38	40	

CG: control group; TG1: afoxolaner treatment; TG2: castor oil treatment; T: time in days; U/L: unit per liter, values above reference.

**Table 3:** Data found in the evaluation of the enzyme alkaline phosphatase (ALP) of all animals of all groups analyzed in the present study at different times of blood collection. Note the reference values for this enzyme on the right of the table.

**Alkaline Phosphatase (ALP)**							
**Group**	**Rabbit**	**T 0**	**T 15**	**T 30**	**T 45**	**T 60**	**Reference value**
**CG**	**1**	301	289	285	298	307	250 – 600 U/L
	**2**	286	341	323	328	295	
	**3**	296	320	336	316	309	
**TG1**	**1**	315	335	327	299	321	
	**2**	321	317	299	276	314	
	**3**	300	319	301	295	289	
**TG2**	**1**	294	288	307	311	296	
	**2**	301	296	279	297	300	
	**3**	297	321	315	319	288	

CG: control group; TG1: afoxolaner treatment; TG2: castor oil treatment; T: time in days; U/L: unit per liter.

By jugular puncture, 2 mL of blood was collected from each rabbit using a 3 mL syringe and 25x0.08 mm needle (sterile) placed in a dry Vacuntainer® with separating gel, for biochemical tests of liver enzymes AST, ALT, and ALP.

## Morphological Analysis

### Obtaining liver and spleen samples from the rabbits

The CG, TG1, and TG2 rabbits were euthanized in a room of the Department of General and Applied Biology Viverium by veterinarian Letícia Maria Graballos Ferraz Hebling, CRMV nº 5.412, using ketamine and xylazine, applied intraperitoneally in dosages of 300 mg/kg and 30 mg/kg, respectively, to collect the fragments of the liver and spleen.

### Harris hematoxylin-aqueous eosin staining (Junqueira & Junqueira, 1983)

The host rabbits’ liver and spleen fragments were fixed in 4% paraformaldehyde for 72 hours at 4 ºC. They were then transferred to a phosphate buffer solution (NaCl 7.5 g/ L, Na_2_HPO_4_ 2.38 g/L, and KH_2_PO_4_ 2.72 g/L), where they remained for 24 hours. Subsequently, they were dehydrated in increasing series of 70%, 80%, 90%, and 95% ethyl alcohol (baths of 30 minutes each), soaked in Leica historesin for 24 hours, and placed in plastic molds containing historesin plus polymerizer (Leica Historesin Kit®). The blocks were sectioned (3 µm thick) in Leica RM2265 microtome (Leica®), the sections were rehydrated for 1 minute in distilled water and then stained by Harris hematoxylin for eight minutes. After washing for three minutes in running water, they were stained by aqueous eosin for five minutes and again washed in running water. After drying, the slides were dipped in xylol and then covered with Entellan and coverslip. The permanent slides were examined and photographed under a Leica DM750 bright field microscope (Leica®).

## Histochemistry

### PAS technique (periodic acid-Schiff) (Junqueira & Junqueira, 1983)

Liver fragments were fixed in 4% paraformaldehyde for 72 hours at 4 ºC and dehydrated in concentrations of alcohol 70%-95% in baths of 30 minutes each to detect neutral polysaccharides. They were soaked in Leica historesin for 24 hours and placed in plastic molds containing historesin plus polymerizer (Leica Historesin Kit®). Later, the blocks were sectioned (3 µm thick) in Leica RM2265 microtome (Leica®), and the sections were rehydrated for 1 minute in distilled water and transferred to a 4% periodic acid solution for 10 minutes. Washing was performed in distilled water for 1 minute and immersion for 1 hour in Schiff's reagent. They were then washed for 15 minutes under running water, dried at room temperature, and mounted on Entellan. The permanent slides were examined and photographed under a Leica DM750 (Leica®) bright-field microscope.

### Bromophenol blue technique (Pearse, 1985)

Fragments of the liver and spleen were fixed in 4% paraformaldehyde for 72 hours to detect total proteins. They were dehydrated in increasing series of ethyl alcohol 70%-95% (30 minutes each), soaked in Leica historesin for 24 hours, and placed in plastic molds containing historesin plus polymerizer (Leica Historesin Kit®). The blocks were sectioned (3 µm thick) in Leica RM2265 microtome (Leica®). The sections were bromophenol blue staining for one hour at room temperature. After being washed under running water for 15 minutes, they were dried, mounted on Entellan, and covered with a coverslip. The permanent slides were examined and photographed under a Leica DM750 (Leica®) bright-field microscope.

## Results

### Clinical analysis

The data obtained from the analysis of blood samples from rabbits from the control groups (CG), the group exposed to afoxolaner (TG1), and the group exposed to esters derived from castor oil (TG2) at times 0, 15, 30, 45, and 60 days are arranged in Table 1 (AST), Table 2 (ALT), and Table 3 (ALP) with the respective results and reference values.

**Table 2:** Data found in the evaluation of the enzyme alanine aminotransferase (ALT) of all animals of all groups analyzed in the present study at different times of blood collection. Note the reference values for this enzyme on the right of the table.

**Alanine Aminotransferase (ALT)**							
**Group**	**Rabbit**	**T 0**	**T 15**	**T 30**	**T 45**	**T 60**	**Reference value**
**CG**	**1**	65	52	47	52	49	34 – 123 U/L
	**2**	43	49	51	47	52	
	**3**	48	56	52	54	45	
**TG1**	**1**	47	49	53	49	50	
	**2**	50	46	48	43	45	
	**3**	52	67	55	45	64	
**TG2**	**1**	59	52	47	54	51	
	**2**	50	47	54	49	44	
	**3**	46	49	44	61	57	

CG: control group; TG1: afoxolaner treatment; TG2: castor oil treatment; T: time in days; U/L: unit per liter.

The data obtained from the enzymes AST, ALT, and ALP of the rabbits allocated in the (CG) served as a reference for comparison with the results obtained from the rabbits allocated in Treatment Groups 1 and 2 (Tables 1-3).

The rabbits allocated to this group were fed with Bandeirante® commercial feed and exposed to two doses of afoxolaner. The first dose was administered at T0 (baseline time) and the second after 30 days at T30. Table 1 shows that this group presented no changes in the parameters of the liver enzymes ALT and ALP. However, it presented a significant increase in serum levels for the enzyme parameter AST at times T15, T30, T45, and T60.

The rabbits (TG2) were fed with the Bandeirante® commercial feed enriched with castor oil ricinoleic acid esters at a concentration of 1.75 g of esters/Kg on days 0, 15, 30, 45, and 60. The blood sample data did not differ from those found in the control group (CG) used as a reference for the enzymes AST, ALT, and ALP.

## Morphology

### Liver-control group (CG)

**Figure 1:**
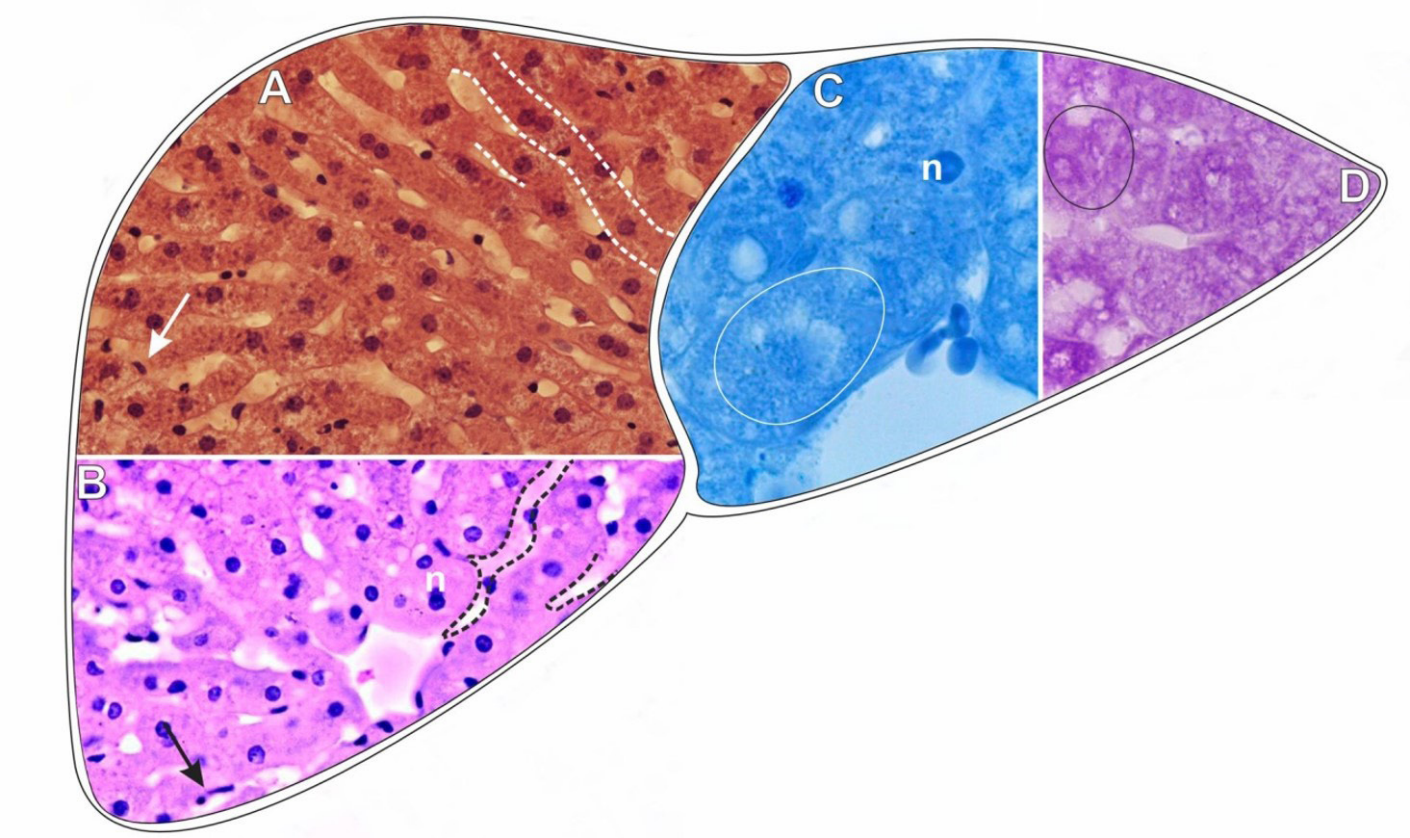
Scheme and histological sections of the liver of rabbits allocated in the control group (CG), stained by HE (A-B) for the general observation of tissue organization and by bromophenol blue (C) and PAS reaction (D) to detect total proteins and carbohydrates, respectively. **Circle**= hepatocyte, **n**= hepatocyte nucleus, **arrow**= Kupffer cell nucleus, **dashed**= hepatocyte cords. **A-B=** 40X. **C-D**= 100X.

The results showed that the liver of the rabbits of the CG, showed preserved liver tissue, with hepatocyte cords parallel to each other and evident cell boundaries. Hepatocytes showed the presence of one or two rounded nuclei and a nucleolus (Figure 1A-1B).

The cytoplasm of the hepatocytes was generally homogeneous, i.e., with no vacuoles or lipid droplets of significant size (Figure 1B).

A large amount of red blood cells was observed inside the hepatic blood vessels, which was also expected.

Nuclei with elongated shapes belonging to Kupffer cells, known as liver macrophages, can be observed between the strands of hepatocytes (Figure 1 A-1B).

Histochemical tests applied to the livers of the (**CG**) individuals confirmed that the liver tissue was preserved (Figure 1C-1D). The test for protein detection showed the cytoplasm nuclei of hepatocytes strongly positive for this element (Figure 1C).

The test for carbohydrates showed hepatocytes with strongly positive cytoplasm since the liver tissue is physiologically involved with the production and storage of complexes with carbohydrates in their constitution (Figure 1D).

The nuclei of hepatocytes and Kupffer cells could not be evidenced due to the specificity of this histochemical technique.

### Liver of afoxolaner treatment (TG1)

**Figure 2:**
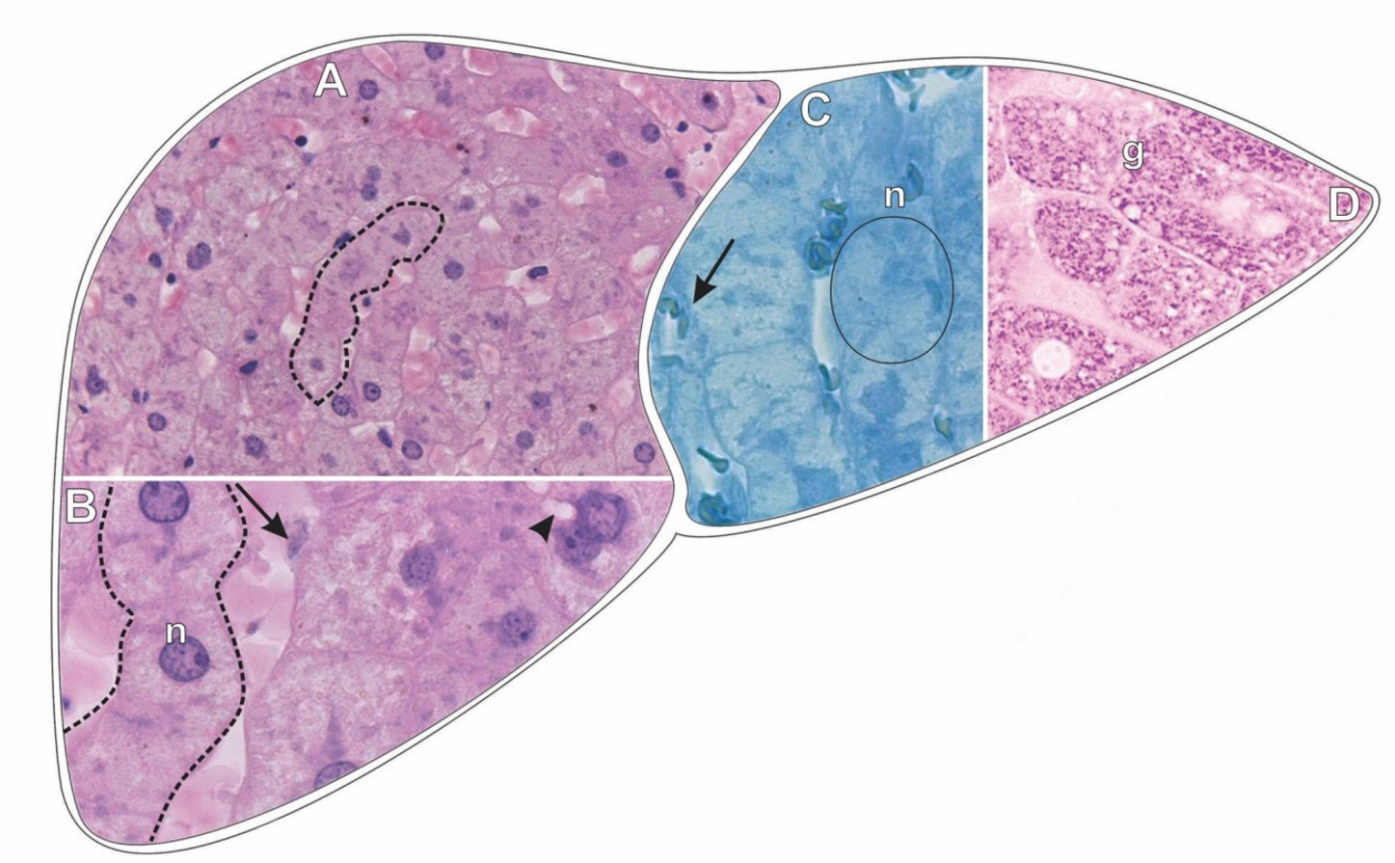
Scheme and histological sections of the liver of rabbits exposed to afoxolaner (TG1) and stained by HE (A-B). Histochemical tests: bromophenol blue (C) and PAS reaction (D) to detect total proteins and carbohydrates, respectively. **Circle**= hepatocyte, **n=** hepatocyte nucleus, **arrow=** Kupffer cell nucleus, **arrow head=** vacuolation around the nucleus, **g=** fine cytoplasmic granulation PAS-positive, **dashed**= hepatocyte cords. **A=** 40X. **B, C, D=** 100X.

Despite showing hepatocytes with their cell boundaries quite evident, the liver tissue of the rabbits exposed to afoxolaner also showed that, unlike what was observed in the rabbits of the (CG), the cytoplasm of these cells was more heterogeneous, that is, with lipid droplets distributed throughout the cell (Figure 2A-2B). The onset of cytoplasmic vacuolation around the hepatocyte nucleus (Figure 2B) and vacuoles between the hepatocyte cords (Figure 2A) were also noted.

Contrary to what was observed in the CG rabbits, the detection of proteins in the liver tissue of the rabbit exposed to afoxolaner was less intense (Figure 2C). The cytoplasm of hepatocytes showed extensive areas with very weak markings or even absence of markings for protein, also highlighting that, contrary to what was observed in the (CG), the hepatocyte nuclei were medially positive (Figure 2C) but still with the evident nucleolus.

Cytoplasmic changes were observed in the PAS reaction for carbohydrate detection compared with the **CG**. Many vacuolated areas could be observed in the cytoplasm of hepatocytes and are composed of strongly positive clusters of fine granulations (carbohydrate aggregates) distributed over almost the entire extent of the hepatocytes (Figure 2D). The nuclei of hepatocytes and Kupffer cells could not be evidenced.

### Liver of castor oil treatment (TG2)

**Figure 3:**
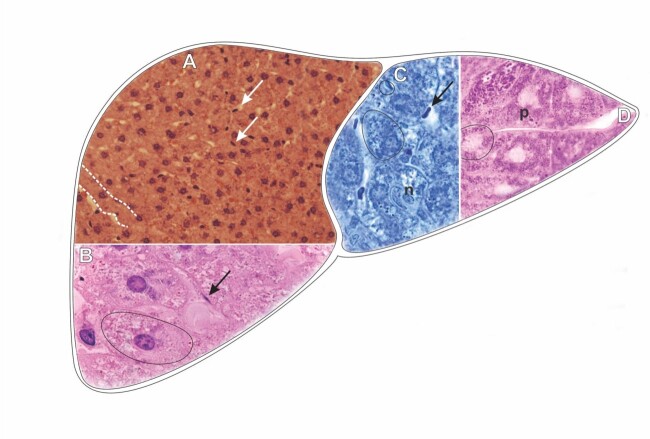
Scheme and histological sections of the liver of rabbits allocated to the castor oil treatment group (TG2) (*Ricinus communis*). The sections were stained by HE (A-B) and bromophenol blue (C), and PAS reaction (D) to detect total proteins and carbohydrates. **Dashed**= hepatocyte cord, **p**= accumulation of polysaccharide granulation, **n**= nucleus, **arrow**= Kupffer cell, **circles**= hepatocyte. **A=** 40X. **B, C, D=** 100X.

Morphological changes in liver tissue were observed in rabbits exposed to Bandeirante® commercial feed enriched with castor oil ricinoleic acid esters. However, these were not as aggressive as those observed in rabbits that received doses of afoxolaner (Figure 3A-3B).

The vacuolation of the cytoplasm concentrated around the nuclei of the hepatocytes in (TG2) was not observed at the time of exposure to esters (Figure 3B). On the contrary, the exposure to esters showed an increase in the form of fine granulation of polysaccharide nature, probably glycogen granules (Figure 3D), showed the cytoplasm of hepatocytes almost filled by strongly positive PAS granulation, contrary to that observed in the exposure of liver tissue to afoxolaner (Figure 2D).

Also under the histochemical aspect, the protein detection in the liver tissue of rabbits exposed to esters showed more significant preservation of elements of a protein nature in the cytoplasm of hepatocytes than was observed in exposure to afoxolaner (Figures 2D, 3D).

Although cytoplasmic vacuolation was evidenced, strong (coarse) granulation of protein elements was observed. Additionally, contrary to what was observed in exposure to afoxolaner, the hepatocyte nuclei were strongly marked by bromophenol blue, as were the nucleoli (Figure 3D).

### Spleen-control group (CG)

**Figure 4:**
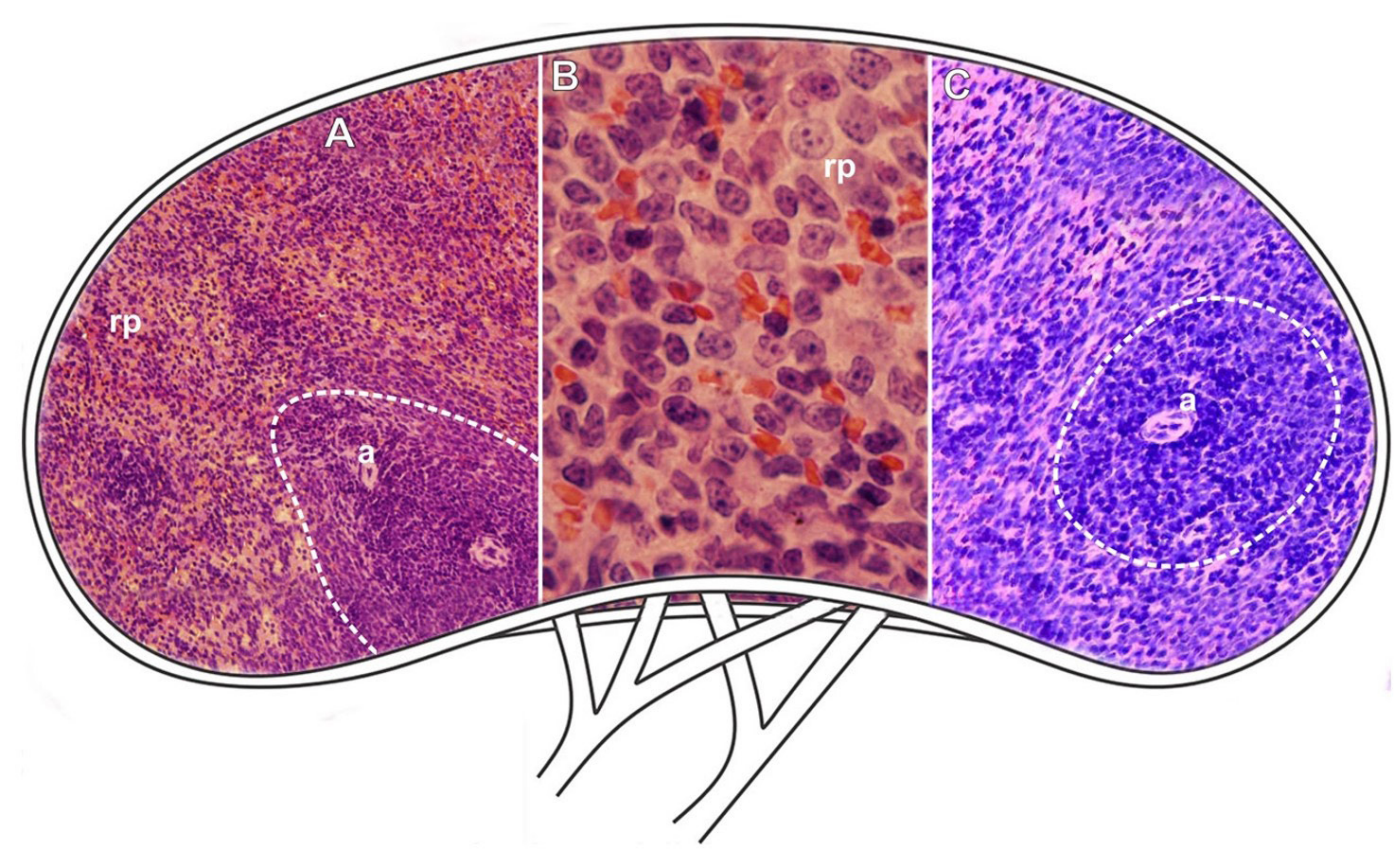
Scheme and histological sections of the spleen of rabbits allocated in the control group (CG), stained by HE (A-C) for the general observation of the tissue after exposure. **Dashed=** white pulp, **a=** arteriole, **rp**= red pulp. **A=** 10X. **B=** 100X. **C=** 40X.

**Figure 5:**
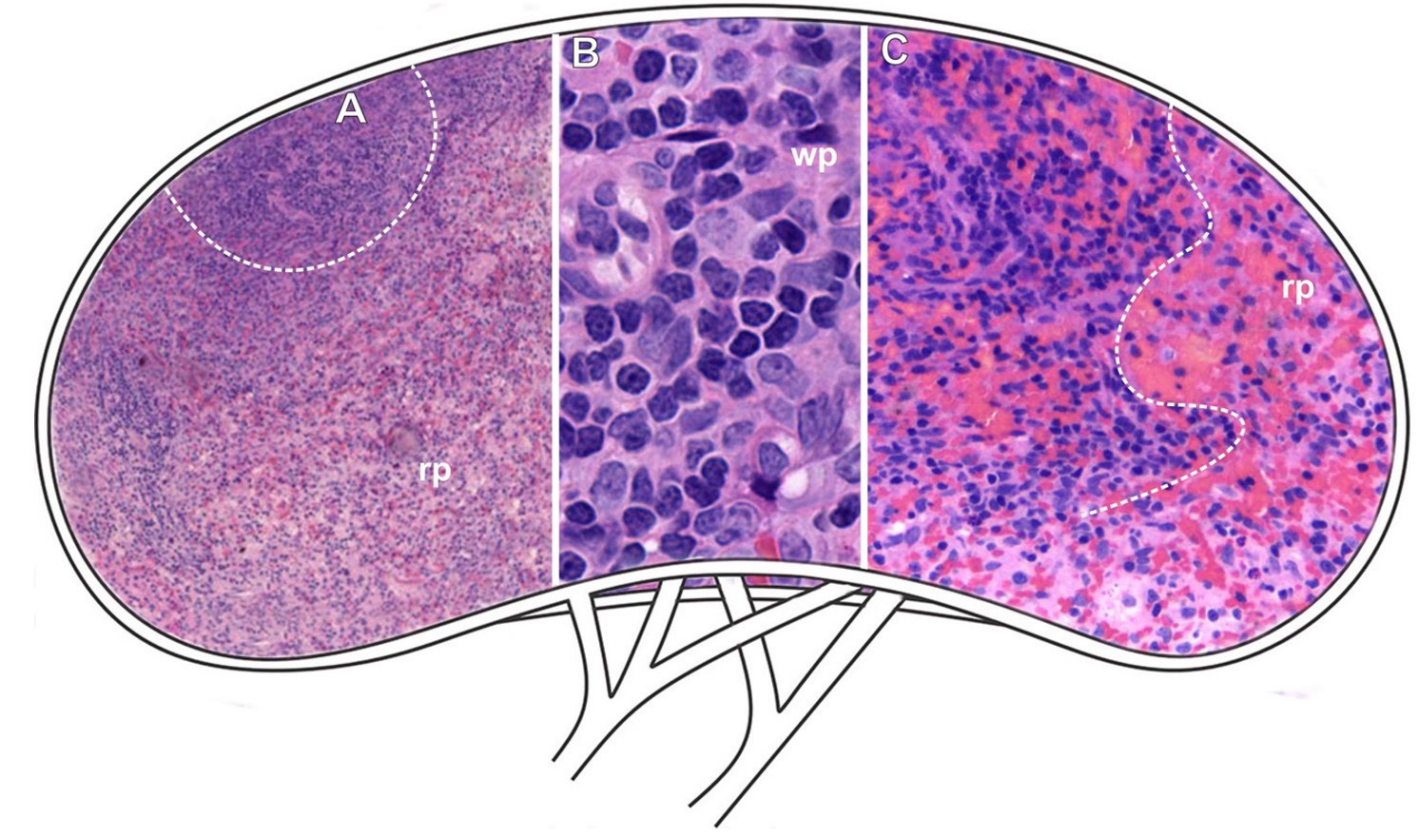
Scheme and histological sections of the spleen of rabbits allocated to the afoxolaner treatment group (TG1), stained by HE (A-C) for the general observation of the tissue after exposure. **Dashed**= white pulp region (**wp**), **rp**= red pulp. **A=** 10X. **B=** 100x. **C=** 40X.

**Figure 6:**
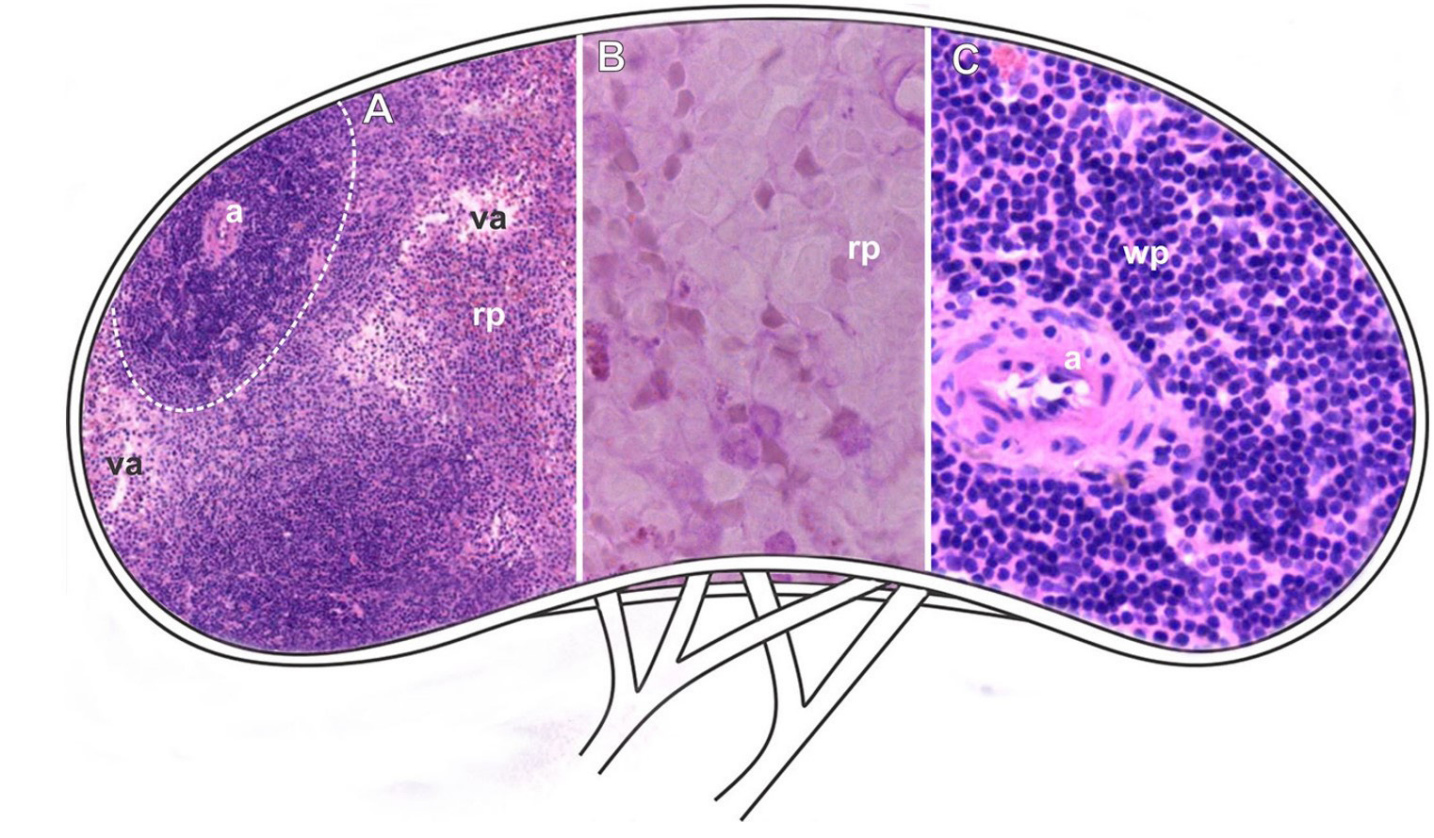
Scheme and histological sections of the spleen of rabbits allocated to the castor oil treatment group (TG2) (*Ricinus communis*), subjected to HE stain (A-C) and PAS reaction (B) to detect carbohydrates. **Dashed**= white pulp region **(wp), a**= central arteriole, **rp**= red pulp. **A=** 10X. **B=** 100X. **C=** 40X.

We also evaluated the morphology/histology of the spleen of rabbits of the control group (CG), afoxolaner treatment (TG1), and castor oil treatment (TG2**)**. The most evident change occurred in the group exposed to castor oil ricinoleic acid esters (TG2) (Figures 4-6), with apparent clear tissue vacuolation when the red pulp was analyzed (Figure 5A).

The spleen is considered the highest concentration of lymphoid tissue in the body, representing an organ of defense against microorganisms and the destroyer of erythrocytes. This organ is subdivided into white pulp (lymphoid tissue) and red pulp (tissue rich in cells and reticular fibers) rich in blood.

**Figure 7:**
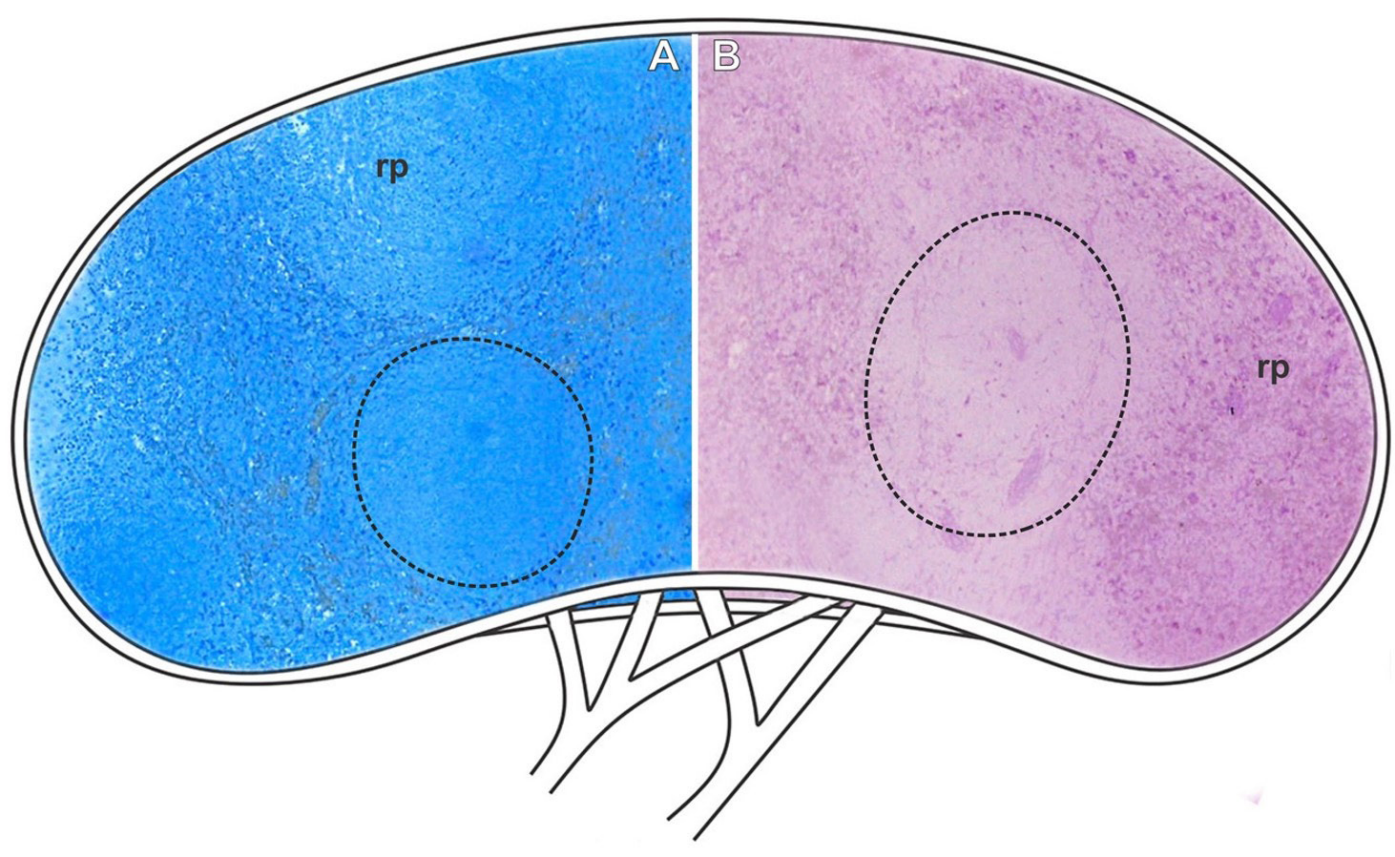
Scheme and histological sections of the spleen of rabbits allocated in the control group (CG), stained by bromophenol blue to detect total proteins (A), and reacted by PAS to detect carbohydrates (B). **Dashed=** white pulp, **rp**= red pulp. **A-B=** 10X.

The data obtained showed that there were only minor morphological changes in the spleen when comparing the two exposure groups (TG1 and TG2) to the control group (CG) (Figures 4-7). 

Histologically, no significant changes were detected when applying the techniques for detecting proteins and carbohydrates (Figure 7A-7B).

## Discussion

Rabbits and dogs share anatomical, physiological, and immunological similarities, making them relevant models for studying the effectiveness of tick control products and strategies in dogs. This approach allows a better understanding of the effects and safety of treatments in a similar biological system. In addition, rabbits are widely used in research and readily available in many laboratories and institutions, facilitating access to these experimental models (Mapara et al., 2012). By using rabbits as models, it is possible to better control the study variables, such as exposure to ticks and the treatments applied, which can be challenging in experiments performed directly on dogs. This approach also reduces the need to expose dogs to invasive or potentially harmful procedures, contributing to a reduction in the use of pets in research. Thus, the use of rabbits in experiments follows the “3Rs” principle (reduction, refinement, and replacement) in animal experimentation, while providing valuable information on the effectiveness of treatments and the understanding of the mechanisms involved in the tick-host interaction (Gad, 2016; Calasans-Maia et al., 2009).

Tick control is necessary to eliminate this ectoparasite, especially in urban environments where hosts, such as dogs, are in constant contact with humans. Because these “pets” are often considered family members, concern about ectoparasite control increases due to the potential for humans to become targets for tick parasitism. Research focusing on the bioactive compounds found in castor oil esters (*Ricinus communis*) have demonstrated their effectiveness in the main organs related to tick feeding (salivary glands) and reproduction (ovaries), as demonstrated by Sampieri et al. (2013), Arnosti et al. (2011), and Sodelli et al. (2022). However, despite the efficiency of esters as acaricides, little is known about their effects on non-target organisms. Many studies, including those by Cunha et al. (2017), Sodelli et al. (2022), and Vieira et al. (2006), highlighted that these compounds can be toxic to both hosts and non-target organisms, despite their potency as acaricides. Synthetic chemical-based acaricides, such as afoxolaner, have gained prominence in the veterinary market. Afoxolaner acts on glutamate receptors, causing excessive neural stimulation and leading to tick death, as described by Drag et al. (2014), Cutolo et al. (2021), and Tinkruejeen et al. (2019). Over all, this research contributes to understanding the challenges and potential risks associated with tick control methods, highlighting the need for further studies to develop safer and more effective strategies for tick management in both human and veterinary contexts.

This information stimulated the development of this project, which aimed to analyze the levels of hepatic enzymes in the liver of rabbits when exposed to natural (castor esters) and synthetic (afoxolaner) acaricides by analyzing the enzymes AST, ALT, and ALP (Tables 1-3), demonstrating that the esters did not change the pattern of these enzymes and the results are within the reference values and close to those of the CG. These findings indicate that esters have a promising safety profile and can be considered a viable option for treating tick infestations. Arnosti et al. (2011) and Sampieri et al. (2013) have proven the effectiveness of these compounds as acaricides, showing their potential in the fight against these ectoparasites, highlighting their relevance in research, and making esters a new control strategy.

On the other hand, the data obtained from rabbits allocated in TG1, exposed to the synthetic acaricide afoxolaner, showed an increase in the parameters of the enzyme aspartate aminotransferase (AST), suggesting the occurrence of drug poisoning, probably due to the synthetic chemical. Aspartate aminotransferase (AST) is a liver enzyme that has its blood levels increased in the presence of liver cell injury. Its specificity is lower than that of ALT, and the increase in serum levels can occur in cases of intoxication, mainly due to drug intake (1 and 2). An increase in this enzyme in the blood of TG1 rabbits was found in the experiment conducted as a result of the drug administration, indicating liver poisoning. However, the blood levels of this enzyme in TG2 remain similar to those of CG, compatible with the reference values indicative of normality. This means that there is no impairment of the liver and that ricinoleic acid esters are more indicated for use as an acaricide.

The analysis of the liver and spleen of rabbits exposed to the synthetic acaricide afoxolaner and the castor oil esters comparatively and from morpho-histological and histochemical aspects showed that the liver of rabbits exposed to afoxolaner presented lipid droplets distributed in the cytoplasm of hepatocytes evidencing the occurrence of hepatic steatosis due to the use of this specific drug. Steatosis is a condition with a significant accumulation of lipids in hepatocytes. Steatosis can be classified as microvesicular (enlargement of the cytoplasmic accumulation of small lipid droplets due to impairment in mitochondrial β-oxidation) or macrovesicular (related to clinical conditions, including nutritional, metabolic, and drug-induced processes) (Minamikawa et al., 2020). The results here presented suggest the installation of macrovesicular steatosis due to exposure to synthetic acaricide. Metabolic disorders can occur due to the prolonged use of substances that cause hepatotoxicity, in this case, the synthetic acaricide, resulting in liver inflammation (Albadry et al., 2022). Excess fat in the cytoplasm of hepatocytes leads to cytoplasmic and tissue disorganization, consequently causing structural changes, especially circulatory, damaging the organ and body since perfect blood circulation is essential for the integrity of liver physiology (Junqueira & Carneiro, 2017). Along with the steatosis observed in the liver of rabbits exposed to afoxolaner, the onset of cytoplasmic vacuolation around the hepatocyte nucleus was noted. Previous work by Cunha et al. (2017) and Roma et al. (2012) showed the occurrence of cytoplasmic vacuolation, including around the nucleus in the liver of mice (*Mus musculus*) due to the exposure to synthetic acaricides fipronil, thymol, and permethrin, which could suggest a strategy of hepatocyte preservation in response to the presence of the toxic element in the tissue. In other words, this vacuolation would be removing damaged organelles or even certain regions of the cytoplasm that were altered by the presence of the toxic element, sheltering them in vacuoles that would later be digested and/or eliminated, thus preserving the content that was still intact in the cytoplasm. The vacuolation around the nucleus could indicate that the liver cell, under the action of the toxic product, would be preserving its genetic material by removing any residue that could alter its DNA, since changes in nuclear physiology, lead to the deregulation of essential characteristics that contribute to the function of hepatocytes (Alberts et al., 2017). The exposure to the drug also showed a vacuolation between the hepatocyte cords, which disorganized the hepatic circulation and suggested a process of tissue edema, which would influence the blood flow through the tissue, thus affecting the efficiency of the organ.

The histochemical evaluation of the liver of rabbits exposed to afoxolaner (TG1) to mark proteins and polysaccharides showed in CG strong positivity for proteins in the cytoplasm of hepatocytes, corroborating Junqueira & Carneiro (2017), who stated that this strong positivity would be normal in hepatocytes since the synthesis and release of proteins would occur in this environment to be later used by the organ or digestive system. Contrary to data from the CG, the rabbits exposed to afoxolaner presented weak positivity for protein. According to Roma et al. (2012), in studies with mice exposed to permethrin, the decrease of protein in the liver due to exposure to the acaricide would signal that the processes involved with protein synthesis and the construction of structural proteins would be impaired, causing damage to the general metabolism of the liver and to the body.

Histochemistry to detect polysaccharides in the liver of rabbits exposed to afoxolaner showed hepatocytes with cytoplasmic areas containing polysaccharide granulation aggregates distributed over almost the entire extent of the cells, data different from those found in the CG, which presented preserved liver tissue, with homogeneous cytoplasm. According to some authors (Cunha et al., 2017; Junqueira & Carneiro, 2017), the increase and accumulation of polysaccharides in the cytoplasm of hepatocytes exposed to toxic products could be the result of processes involved with the elimination of metabolites, in this case, the afoxolaner.

The morpho-histology and histochemistry of the liver of rabbits exposed to the castor oil ricinoleic acid esters (TG2) were also analyzed. The results showed that, although of natural origin, they still caused changes in liver tissue, but less aggressively than the rabbits exposed to the synthetic drug. The liver of rabbits exposed to esters showed hepatocytes organized in hepatic cords and remained intact since their cellular outlines could be evidenced. The cytoplasm of these cells remained as in the CG without the presence of lipid droplets. This same pattern was described in the study of Shanmuganath et al. (2021), who reported the absence of hepatocellular alterations in rats exposed to the natural bioactive compound *Ageratum conizoydes.* The hepatocytes of rabbits of TG2 presented well-preserved nuclei and nucleoli indicating that esters did not cause toxicity to cells, since that changes are directly linked to the occurrence of cell death, a cellular strategy to preserve the tissue. Exposure to esters did not cause cytoplasmic or nuclear vacuolation in hepatocytes, also absent or minimal between the strands of hepatocytes, indicating that the esters at the concentration in question were not hepatotoxic (Gonzalez & Silva, 2017).

The liver rabbits exposed to castor oil esters showed strong PAS positivity in the cytoplasm of hepatocytes due to an increase in the intensity of polysaccharide granulation, most likely an accumulation of glycogen. According to the literature, this suggests that the esters somehow interfered in the production and storage of this element when compared with the CG and TG1. Glycogen, abundant in hepatocytes shows the individual’s nutritional status (Gonzalez & Silva, 2017), or the presence of toxic substances in the system. In this case, the data suggested that esters stimulated glycogen production.

The observations regarding proteins indicated no change in the histochemical pattern when compared with the data obtained in the CG, demonstrating that the liver continued to synthesize and release/store proteins, even in the presence of esters, corroborating the morphological information described for the mammalian liver (Junqueira & Carneiro, 2017).

The spleen here analyzed, in addition to other functions, is involved with immunology, acting against the presence of antigens that may be transported via blood circulation (Santos, 2017). Morphologically, it is divided into red pulp (splenic cords where blood capillaries, red blood cells, macrophages, dendritic cells, and stromal cells) and acts in blood filtration, destruction of erythrocytes, and elimination of immune complexes, and by the white pulp (B and T lymphocytes, macrophages, and dendritic cells) responsible for processing the antigens transported by the bloodstream.

The spleen analysis revealed no changes in the tissue of rabbits exposed to afoxolaner (TG1) since the same morphological characteristics found in the control group were observed. The exposure to castor oil esters showed vacuolation, not cellular, but tissular of the red pulp, data not found in other studies with spleen exposure to other bioactive compounds. The histochemical techniques to detect proteins and polysaccharides in this organ showed results similar to those found in CG, i.e., the absence of changes due to exposure to synthetic and natural acaricides.

In conclusion, the data obtained in this study revealed that the castor oil esters caused no detectable toxicity in the rabbits exposed to them when incorporated into the feed, data confirmed by clinical analysis of the hepatic parameters of the enzymes. The morphological data of the liver and spleen showed that the castor oil esters did not induce detectable alterations the cells or tissue of these organs indicating that esters may be an alternative for tick control that causes minimum damage to the hosts and is potentially cleaner due to its lower contamination compared to synthetic chemical acaricides that leave toxic residues in the environment. On the other hand, the administration of afoxolaner resulted in a significant increase in the AST enzyme, indicating an alleged occurrence of drug poisoning in rabbits exposed to it. In addition, morphohistological changes in the liver were observed, including the presence of steatosis (accumulation of fat) and cellular and tissue vacuolizations. These findings corroborate the hypothesis that afoxolaner can cause liver damage in cases of improper administration.
